# Misleading concerns about computed tomography scans

**DOI:** 10.1093/jncics/pkaf090

**Published:** 2025-10-27

**Authors:** Drew Moghanaki, Ashley E Prosper, Denise R Aberle

**Affiliations:** Department of Radiation Oncology, David Geffen School of Medicine at UCLA, Los Angeles, CA, United States; Department of Radiology, David Geffen School of Medicine at UCLA, Los Angeles, CA, United States; Department of Radiology, David Geffen School of Medicine at UCLA, Los Angeles, CA, United States

A recent article by Smith-Bindman et al. published by *JAMA Internal Medicine* on April 14, 2025, with a headline of “Original Investigation | Less Is More” projected that up to 5% of future cancers in the United States may result from computed tomography (CT) imaging;^1^ this has understandably alarmed the public. Although vigilance regarding radiation exposure is important, these projections were based solely on theoretical models—not clinical evidence—and risk provoking undue fear among patients and providers.

The authors used a linear no-threshold model, which while widely used, has known limitations.^2^ They assumed uniform cancer risk across all patients, which is an implausible assumption. For example, a critically ill patient undergoing CT for diagnosis or treatment should not be equated with a healthy person receiving low-yield imaging. Such broad generalizations reduce clinical relevance and can deter necessary scans as a result of unwarranted fears.

When used appropriately, CT imaging is a cornerstone of modern medicine. In our practice, it improves diagnostic accuracy, guides curative treatment, and prevents unnecessary procedures. Low-dose chest CT (LDCT) lung cancer screening, for example, prevents premature deaths from lung cancer in high-risk individuals^3^ and delivers an average radiation dose of 2 mSv^4^—comparable to several months of background exposure.

When evaluating the randomized trials confirming the benefits of LDCT for lung cancer screening, the benefits far outweigh the associated radiation risks as reported by Hendrick and Smith.^5^ In the National Lung Screening Trial, the estimated ratio of lives saved to radiation-induced cancer deaths was approximately 12:1 for men and 19:1 for women when considering only the screening dose, with a combined overall ratio of 16:1. When factoring in both screening and estimated follow-up imaging doses, the ratio remained strongly favorable—9:1 for men, 13:1 for women, and 12:1 overall. Similar findings were reported in European randomized lung screening trials, including a benefit-to-risk ratio as high as 63:1 in the ITALUNG study and approximately 23:1 in the COSMOS trial.

Importantly, the article offers no discussion of the diagnostic or therapeutic benefits of CT, including its established role in detecting early-stage malignancies, monitoring disease progression, identifying comorbid conditions, and reassuring patients when disease is absent. We also note the potential conflict of interest that was disclosed by the study’s lead author, who is a cofounder of a company marketing radiation dose-tracking software. This high level of transparency regarding financial interests is critical, particularly when research publications in major academic medical journals could influence public perception of commercially available medical technologies.

Moving forward, it is imperative that the medical community remain diligent in scrutinizing modeling-based publications that may alarm the public and undermine confidence in essential diagnostic imaging tools. Efforts to reduce unnecessary radiation are commendable but must be balanced against the life-saving benefits of appropriate CT use. We believe that this principle should guide both research and editorial policy across the medical field.

## Data Availability

No new data were generated or analyzed for this editorial.
